# MicroRNA-194-5p Levels Decrease during Deep Hypothermic Circulatory Arrest

**DOI:** 10.1038/s41598-018-32426-x

**Published:** 2018-09-19

**Authors:** Xiaohua Wang, Zerong You, Guoguang Zhao, Tianlong Wang

**Affiliations:** 1 Department of Anesthesiology, Xuanwu Hospital, Capital Medical University, Beijing, 100053 China; 2 Institute of Geriatrics, Beijing, China; 3 National Clinical Research Center for Geriatric Disorders, Beijing, China; 4 Department of Neurosurgery, Xuanwu Hospital, Capital Medical University, Beijing, 100053 China; 5 Department of Anesthesiology, MGH, Harvard medical school, Boston, USA

## Abstract

Hypothermia has been reported to be effective in protecting the brain in various clinical conditions, including resuscitation after cardiac arrest and complex cardiovascular surgery, and is considered to be a promising therapy for stroke. The present study aimed to confirm the pivotal role that miRNA-194-5p plays in deep hypothermia circulation arrest. On the basis of reductions in expression of miR-194-5p in the circulation of 21 aortic dissection patients who underwent deep hypothermia circulatory arrest, the specific expression, target, and function of miR-194-5p was investigated using primary neuron culture, polymerase chain reaction, *in situ* hybridization, and flow cytometry methods. Our results showed that miR-194-5p expression was significantly downregulated in hypothermia oxygen glucose deprivation-treated neurons *in vitro*. Cortical neurons transfected with miR-194-5p mimic exhibited increased death due to oxygen-glucose deprivation. MiR-194-5p mediated the regulation of neuronal death, which involves the downregulation of the specific target protein SUMO2, which is crucial to ischemia tolerance. Collectively, these data highlight the unique role of miR-194-5p in mediating the deep hypothermia circulation arrest response via the regulation of SUMO2. These findings suggest that miR-194-5p could be a potential therapeutic target for intervention in ischemic disease.

## Introduction

Deep hypothermic circulatory arrest (DHCA) has long been practiced in the repair of aneurysms of the thoracic aorta, giant intracranial aneurysms in adult patients, and complex congenital heart disease in neonates^1–5^. Although the potential of deep hypothermia to protect organs from ischemia damage is well established, little is known about the mechanisms underlying organ protection, or strategies to maximize its efficacy. Elucidating the mechanisms underlying the protection of organs using deep hypothermia is, therefore, of tremendous clinical interest. Understanding these mechanisms would be a pivotal step toward designing therapeutic strategies to activate these processes and, thus, induce a state of tolerance to transient ischemia without risking the occurrence of adverse events associated with deep hypothermia.

MicroRNAs (miRNAs) are short, non-coding RNAs that serve as translation inhibitors through promotion of messenger RNA (mRNA) degradation by imperfect base-pairing between the seed region in miRNA and the miRNA binding site in the 3′-untranslated region (3′ UTR) of target mRNAs. miRNAs can potentially target hundreds of distinct mRNAs and have been increasingly recognized as key regulators of gene expression involved in neuronal protection^6–8^. Surprisingly, miRNAs withstand repetitive freeze-thaw cycles, blood haemodialysis, and are even resistant to nuclease digestion^9^. Due to size, tissue specificity, and inherent molecular stability, microRNAs afford the opportunity to investigate potential new treatments to activate endogenous neuroprotective pathways before performing surgical procedures that require a period of circulatory arrest, increasing the resistance of neurons to a transient interruption in blood supply.

The present series of experiments was designed to demonstrate changes in miR-194-5p expression after DHCA in patient blood. We also tested miR-194-5p expression after hypothermic oxygen-glucose deprivation (OGD) in neurons. To further investigate the important role of miR-194-5p in DHCA, miR-194-5p mimics were transfected into neurons, which lead to increases in neuronal death in OGD-triggered neurons mediated by the downregulation of small ubiquitin-like modifier (SUMO2). This study aimed to provide new insights into the understanding of DHCA, suggests that miR-194-5p plays a key role in DHCA and, furthermore, may have important therapeutic applications in ischemic conditions.

## Results

### Significant decreases in MiR-194-5p levels after DHCA compared with preoperative levels *in vivo*

**Figure 1:**
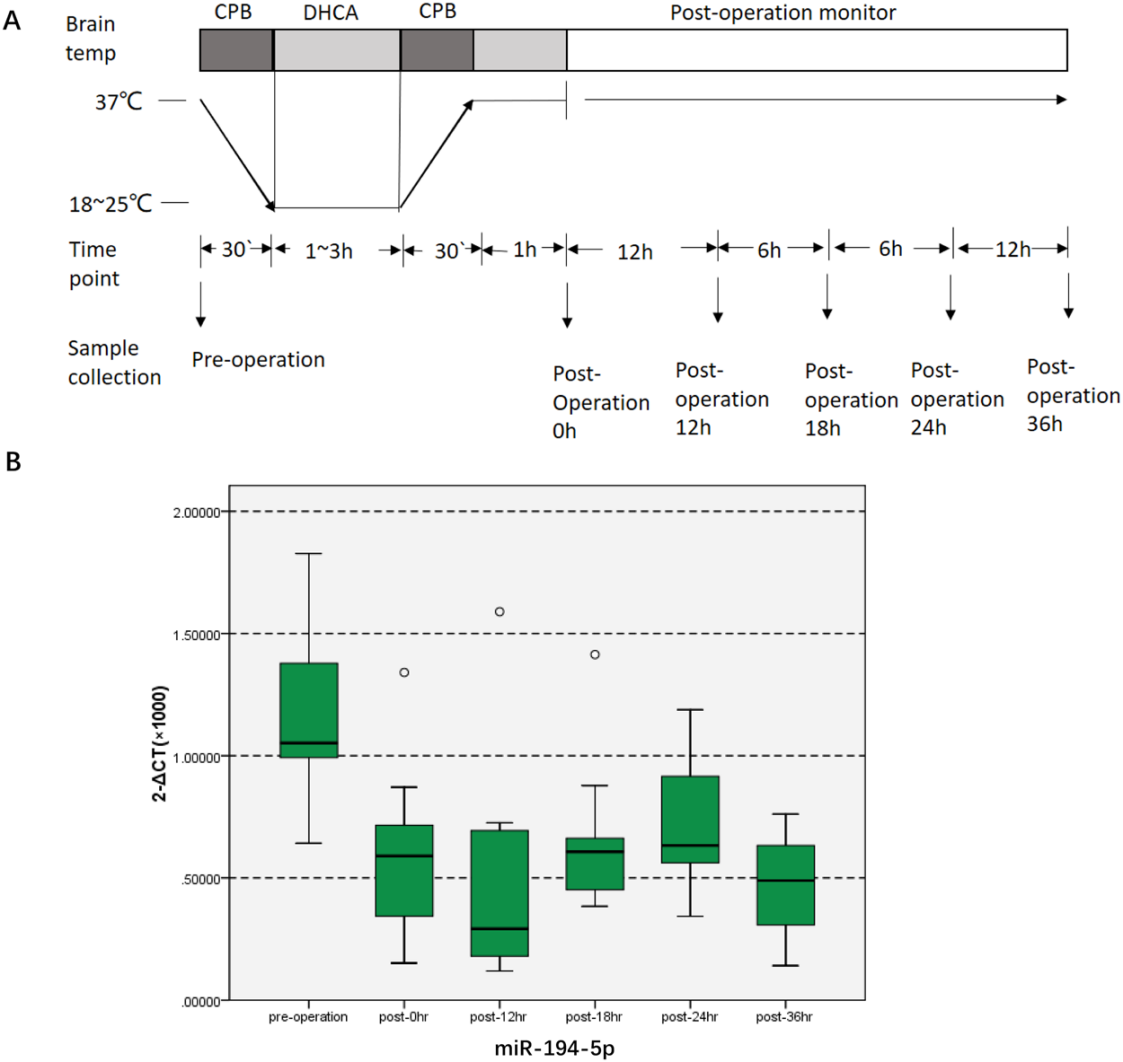
miR-194-5p expression decreases in human blood after DHCA. (**A**) Diagrams illustrating the DHCA procedure, the blood samples were collected and miR-194-5p were analyzed preoperatively, and at 0 h, 12 h, 18 h, 24 h, and 36 h postoperatively. (**B**) Profile of miR-194-5p expression in human blood at different time points. The 2^−ΔCT^ (×10^3^) value of miR-194-5p at 12 h postoperatively was significantly lower than preoperative levels (0.6699 ± 0.3896 versus 1.2629 ± 0.3622; *P* = 0.005). MiR-194-5p levels in the blood 12 h postoperatively were nearly one-half of preoperative levels, remained low level 36 h after the operation (*P* < 0.0001). Compared with postoperative 18 h and 24 h, the expression of miR-194-5p was lower at 12 h postoperatively.

The present study investigated patients undergoing total arch replacement surgery with DHCA. Coincident with results reported previously, miR-194-5p is downregulated in the hippocampus after DHCA in the piglet model (Supplementary Table). The baseline characteristics of these patients and their intraoperative data are summarized in Table 1. Blood samples were collected, and miR-194-5p was analysed at a preoperative time point, then at 0 h, 12 h, 18 h, 24 h, and 36 h postoperatively (Fig. 1A). Expression of miR-194-5p in blood obtained from patients undergoing DHCA was determined by using quantitative real-time polymerase chain reaction (qRT-PCR). It was confirmed that the expression of miR-194-5p significantly decreased after DHCA, declining to the lowest level at 12 h postoperatively. The 2^−ΔCT^ (×10^3^) values of miR-194-5p at 12 h postoperatively were significantly lower than the preoperative values (0.6699 ± 0.3896 *vs* 1.2629 ± 0.3622, respectively; *P* = 0.005). Blood levels of miR-194-5p at 12 h postoperatively were almost one-half of preoperative levels and remained at a low level until 36 h postoperatively (*P* < 0.0001). Compared with 18 h and 24 h postoperatively, the expression of miR-194-5p was also lower at 12 h postoperatively (*P* = 0.012, *P* < 0.0001, respectively) (Fig. 1B). No patients died during hospitalization.

**Table 1:** Demographic and per-operative data.

Variables	Means ± standard deviation
Age (y)	52.7 ± 9.3
Weight (kg)	69.9 ± 12.7
BSA (m^2^)	1.80 ± 0.2
EF (%)	59.4 ± 6.1
Operation time (h)	6.80 ± 1.5
CPB time(min)	188.0 ± 48.5
Cooling time(min)	67.6 ± 30.3
Rewarming time (min)	120.7 ± 27.6
Aortic cross clamp time(min)	94.5 ± 29.1
DHCA time(min)	21.4 ± 9.3
Nasopharyngeal temperature (°C)	19.8 ± 2.4
Ventilation time (h)	18.9 ± 11.7
ICU length of stay (h)	107.8 ± 128.9
Hospital length of stay (day)	12.6 ± 3.2

The study was conducted with patients undergoing total arch replacement surgery under deep hypothermic circulatory arrest. The baseline characteristics of those patients and intraoperative information are summarized.

All the values are given using mean ± SD. BSA: body surface area; EF: ejection fraction; DHCA: Deep hypothermic circulation arrest; CPB: Cardiopulmonary bypass; ICU: Intensive Care Unit.

### MiR-194-5p expression is significantly reduced in neurons subjected to OGD under deep hypothermia *in vitro*

**Figure 2:**
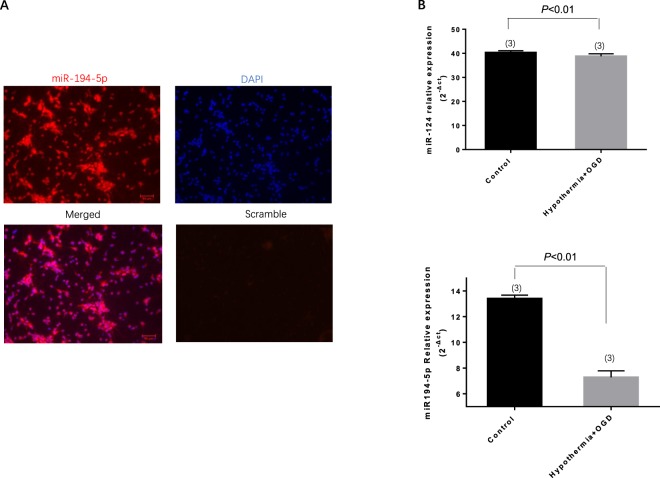
miR-194-5p expression in normal neurons and in neurons subjected to hypothermia and oxygen-glucose deprivation (OGD) conditions. (**A**) miR-194-5p expression is enriched in primary cortical neurons in fluorescence situ hybridization. (**B**) miR-124 and miR-194-5p expression profiles in primary neurons after hypothermia OGD according to quantitative real-time polymerase chain reaction assay. *In vitro*, miR-124 expression which exhibited no significant change after hypothermia OGD. However, miR-194-5p levels decreased significantly after hypothermia OGD.

Initially, the high expression of miR-194-5p was demonstrated in primary rat neurons using locked nucleic acid (LNA) fluorescence *in situ* hybridization (LNA-FISH) (Fig. 2A). Cell culture models of ischemia, in which hypothermia was combined with OGD in neurons, have shown specific changes in miR-194-5p and miR-124, which have been confirmed as brain-specific microRNAs. As expected, primary neurons exhibited decreased miR-194-5p expression after hypothermia OGD (*P* *< *0.01); however, miR-124 demonstrated no significant change after hypothermia OGD (*P* > 0.05) (Fig. 2B).

### MiR-194-5p mimic mediates *increases* in neuronal death after OGD and re-oxygenation

**Figure 3:**
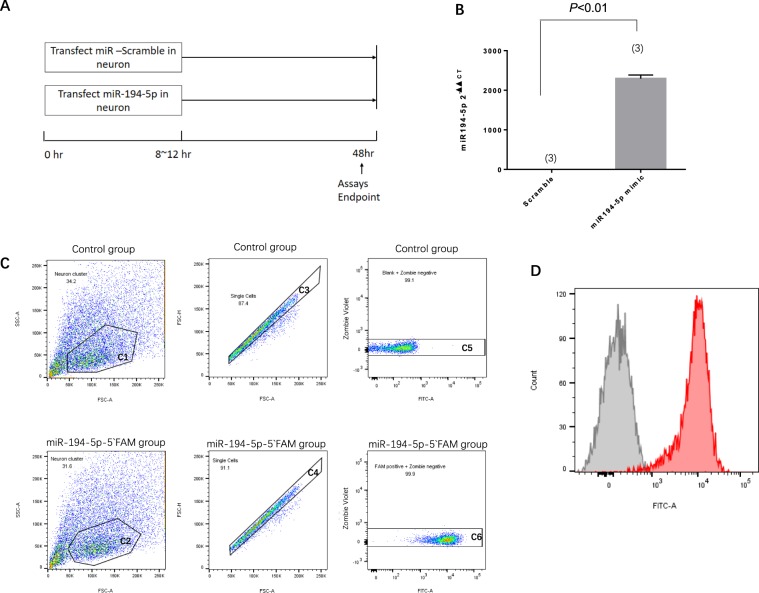
MiR-194-5p transfection and upregulation in neurons. Seven-day-old primary cortical neurons were transduced with scramble, miR-194-5p mimic, were lysed 48 h later. (**A**) Diagrams illustrating the transfection and sample collection procedure. (**B**) The extent of miR-194-5p overexpression efficiency was assessed using quantitative real-time polymerase chain reaction assay. Compared with scramble transduced cells, neuronal cultures transduced with miR-194 mimic exhibited an approximately 2000-fold increase of miR-194-5p expression. (**C**) Transfection efficiency in primary neurons confirmed by fluorescence activated cell sorting analysis. Both two groups exhibit Zombie-violet near 0 (Neuron survival rate >99%; transfection efficiency >97%). Control group: Blank + Zombie negative = 99.1%; FAM positive + Zombie negative = 99.9% (**D**) Histogram plot: the miR-194-5p-5′FAM with higher FITC intensity appear to the right on the x-axis (Red colour), the peak value was at 10^4^. The control group with lower FITC intensity appears to the left on the x-axis (Gray colour), the peak value was near 0. That represent that the miR-194-5p-5′FAM transfected effectively into the living neurons compare with no transfect control group.

**Figure 4:**
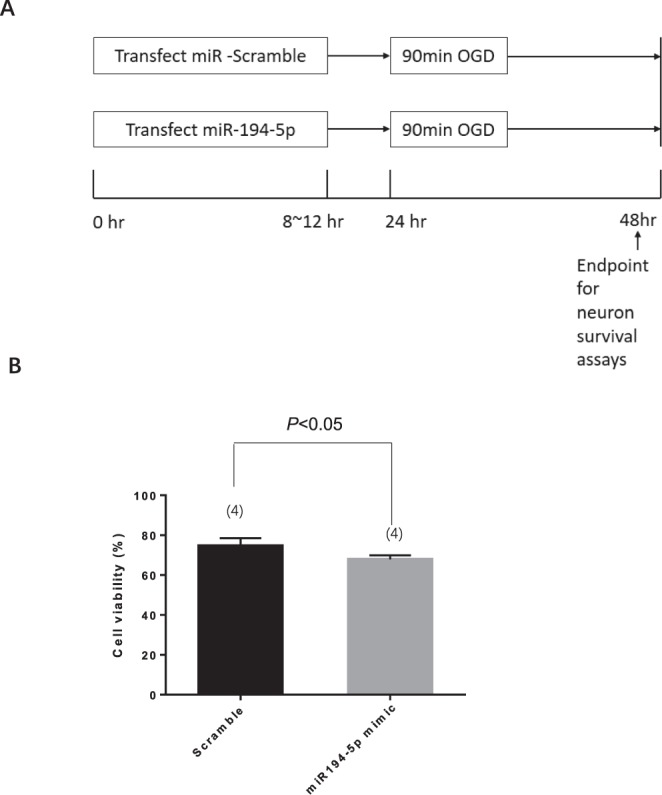
MiR-194-5p mimic decreased neuron viability after ischemia and re-oxygenation. (**A**) Diagrams illustrating the transfection and sample collection procedure. (**B**) Cell viability of primary neurons after oxygen-glucose deprivation in scramble group and miR-194-5p mimic group.

To evaluate the possible effects of miR-194-5p, 7-day primary cortical neurons were transduced with miRNA scramble or miR-194-5p mimic (Fig. 3A). It should be noted that irrespective of the transduction scramble, the miR-194-5p mimic had no overall effect on neuronal survival, compared with the no-transduction neuron group; neuron survival rate >99% (Fig. 3C). Transduction efficiencies were >97%, as analysed by FACS analysis (Fig. 3C). The extent of miR-194-5p overexpression after miR-194-5p mimic transfection was assessed using a qRT-PCR assay. Compared with the scramble miRNA transduced control cultures, neuronal cultures transduced with miR-194-5p mimic exhibited an approximately 2000-fold increase in miR-194-5p expression (Fig. 3B). Eighteen hours post-transduction, cortical neurons were exposed to OGD and then kept in normal culture for an additional 24 h. Neuronal viability was then monitored by using the Cell Viability Assay (Fig. 4A). OGD for 90 min led to a significantly greater increase in neuronal death in the miR-194-5p mimic group, compared with the scramble group (Fig. 4B).

### MiR-194-5p regulates SUMO2 expression in neurons

**Figure 5:**
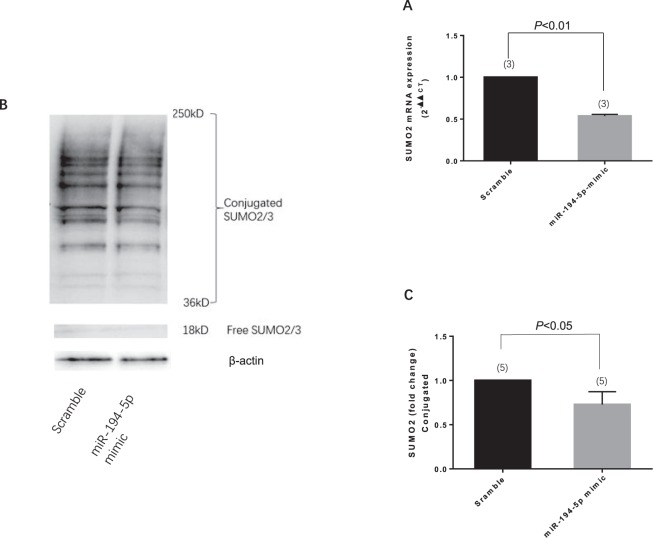
MiR-194-5p controls SUMO2 production in primary rat neurons. (**A**) Diagram for miR-194-5p produce procedure and binding site at SUMO2 3′UTR leading SUMO2 translation repression and degradation. (**B**) Primary rat neurons were transfected with miRNA scramble and miR-194-5p mimics. After 48 h, cells were harvested, and the SUMO2 mRNA expression levels were evaluated using real-time polymerase chain reaction. Relative expression of miR-194-5p mimics was 0.5369 ± 0.0205, compared with the scramble group (*P < *0.01). (**C**) SUMO2 protein level was assessed by western blotting. The fold change was 0.7278 ± 0.1448 in miR-194-5p mimics group compare with scramble group (*P < *0.05). Scramble group represents the meaningless oligonucleotide transfection; it was the control for miR-194-5p mimics.

**Figure 6:**
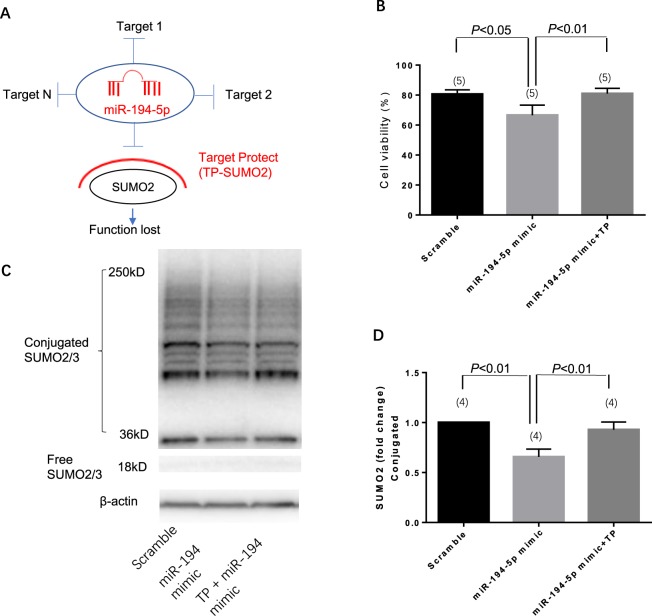
miR-194-5p mimic co-transfected with SUMO2 target protect (TP- SUMO2) demonstrates that SUMO2 is the specific target of miR-194-5p. (**A**) Diagram illustrating the TP-SUMO2 function. (**B**) The band and blots of SUMO2 protein. Compared with miR-194-5p scramble, the SUMO2 expression of miR-194-5p mimic was 0.6563 ± 0.078 (*P < *0.01); miR-194-5p mimic co-transfection with TP-SUMO2 was 0.9275 ± 0.078 (*P < *0.01).

**Figure 7:**
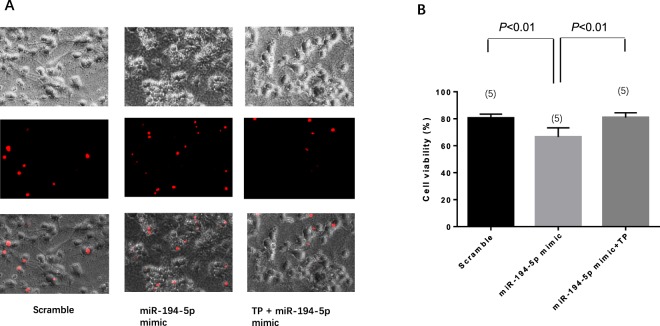
miR-194-5p mimic co-transfected with SUMO2 target protect (TP) demonstrates that SUMO2 is the independent functional target of miR-194-5p. (**A**) Propidium iodide (PI) staining show TP-SUMO2 functionally reverse the neuron death back to the level of the control group. (**B**) Cell viability after transfection of scramble was 80.5569 ± 2.9359, miR-194-5p mimic was 66.5388 ± 6.7488 (*P < *0.01), and miR-194-5p mimic co-transfection with TP-SUMO2 was 80.9443 ± 3.4828 (*P < *0.01).

The next step was to predict the target of miR-194-5p by using the target scan algorithm. Small ubiquitin-related modifier-2/3 (SUMO-2/3) is a member of the ubiquitin-like protein family that has a conserved miR-194-5p binding site within its 3′-UTR in most species; it has been identified as the putative target of miR-194-5p (Fig. 5A). Transfected miR-194-5p mimic downregulated the expression of both SUMO2 mRNA (Fig. 5B) and protein (Fig. 5C) in primary rat neurons. To further confirm whether the effect of miR-194-5p on SUMO2 was direct and specific, primary neurons were co-transfected with miR-194-5p mimic in the presence of a target protector oligo (TP-SUMO2) that specifically protected the miR-194-5p binding site on the endogenous SUMO2 3′-UTR. As shown in Fig. 6b, in the presence of TP-SUMO2, miR-194-5p mimic failed to downregulate the expression of SUMO2. Consistent with this finding, transfection of primary neurons with the TP-SUMO2 specific for the miR-194-5p binding site resulted in amelioration of miR-194-5p mimic-mediated induction of neuronal death (Fig. 7A,B).

## Discussion

In summary, our *in vivo* and *in vitro* studies demonstrated the following: MiR-194-5p levels significantly decreased in human blood after DHCA *in vivo* and in neurons under hypothermia OGD *in vitro*; upregulated miR-194-5p decreased neuronal viability after oxygen and glucose deprivation and re-oxygenation, and SUMO2 was a functional and specific target of miR-194-5p in neurons.

This study is the first to report that miR-194-5p expression decreases in the blood of patients undergoing DHCA. Moreover, the miR-194-5p expression profile in hypothermic neurons exposed to ischemia *in vitro* surprisingly coincided with our previous results using hippocampi from piglets after DHCA. Previously, we reproduced the DHCA model in piglets; harvested hippocampi underwent miRNA microarray analysis using significance analysis of microarrays, and this result was further confirmed by the qRT-PCR assay. Thirty-five miRNAs were differentially expressed in the hippocampus after the DHCA procedure: 13 were upregulated, 22 were downregulated, and miR-194-5p levels decreased significantly in the DHCA group (Supplement-table)^10^. More recently, gene expression in the hippocampus after DHCA has been reported to play a critical role in the pathogenesis of neurological injury^7,11^. Compared with other DHCA pathological mechanisms, much less is known about regulation at the post-transcriptional level in cerebral ischemia after DHCA.

Additionally, for the first time, we revealed that the expression of miR-194-5p can directly upregulate SUMO2 production at the post-transcriptional level. SUMO-2/3 conjugation was involved in the protective effects induced by deep hypothermia and may play a role in DNA repair, transcription regulation, and subcellular localization^12–14^. The profound reduction in body temperature would be lethal in most mammalian species. During the torpor phase in hibernating animals, brain SUMO2 conjugation and levels of SUMO2 proteins exhibit a marked increase, which indicates the presence of a protective response shielding neurons from damage induced by low blood flow and substrate deprivation^15^. A series of studies verified that global increases in SUMO2 and activation of SUMO2 conjugation at the same time are endogenous neuroprotective stress responses to severe stress^16–18^. Our results, which demonstrated a significant decrease in the levels of SUMO2/3-conjugated proteins after upregulation of miR-194-5p expression in neurons, support the concept that elevated miR-194-5p expression and downregulated SUMO2 levels could inhibit a protective stress response. Using the Target-Protect SUMO2 (TP-SUMO2), we further verified that SUMO2 is functionally and specifically downstream of miR-194-5p.

Nevertheless, there are several issues that warrant further consideration. First, astrocytes, oligodendrocytes, and endothelial cells involved in the neurovascular unit may also be targets of miR-194-5p and participate in a hypothermia protection mechanism, which warrants further study. Second, miR-194-5p may be also involved in the potential protection of hypothermia under ischemia. Subsequent steps of this study will use transgenic mice to test the miR-194-5p function *in vivo* in a DHCA model, and a subsequent preclinical study. Third, whether miR-194-5p expression levels truly correlate with long-term outcomes of the patients undergoing DHCA remains to be fully assessed. Lastly, we will design moderate hypothermia groups at different temperatures in animal studies, to test whether different degrees of hypothermia affect the expression of miR-194-5p.

Collectively, the results of our study provide new insights into the role of miR-194-5p in human blood undergoing DHCA and via hypothermia OGD *in vitro*. MiR-194-5p mimic increases neuronal death linked to OGD. These data suggest a unique mechanism of miRNA and hypothermia protection efficiency via post-transcriptional regulation. Modulation of miR-194-5p/SUMO2 can be envisioned as a potential therapeutic strategy for ischemic brain injury.

## Methods

### Study population, operation process, and blood sample collection

This study was a randomized single-blind prospective cohort trial. It was registered at http://www.ClinicalTrial.com. (ClinicalTrial-NCI-03339414) (https://clinicaltrials.gov/). The use of human blood in our study (ID 2013-026283-1) was approved by the institutional review board (IRB) of Xuanwu Hospital (No. Xuanwu 130-63528), and informed consent was obtained from each subject. Furthermore, the studies were conducted as outlined in the IRB protocol. This case-control study included 21 aortic dissection patients who were classified according to the Stanford classification: Stanford A1 (n = 13); A2 (n = 4); A3 (n = 1); B1 (n = 1); and B3 (n = 1); and aortic dilation (n = 1). Patients received standard perioperative monitoring and conventional intravenous inhalation anaesthesia. A median sternotomy was performed, an arterial cannula was inserted into the right axillary artery, and a dual-stage atriocaval cannula was placed in the right atrium, as routinely established for cardiopulmonary bypass. Cardiopulmonary bypass flow rate was maintained between 2.5 and 2.8 L/min/m^2^. During the cooling phase, the antegrade perfusion of cold-blood cardioplegia solution was directly infused into the coronary ostia. Circulatory arrest was established when the nasopharyngeal temperature reached 18–20 °C. An alpha-stat acid-base management strategy was used while nasopharyngeal temperatures were ≥28 °C; a pH-stat acid-base management strategy was used throughout the DHCA procedure when the nasopharyngeal temperature was <18 °C. The carotid artery and subclavian artery were clamped, and antegrade selective cerebral perfusion commenced via the right axillary artery at rate of 10 mL/kg/min. The details of these procedures have been described previously^19^. Blood samples were drawn preoperatively, and at 0, 8, 12, 24, 36 h postoperatively from all patients. Whole blood samples (5–10 mL) were collected into sodium EDTA tubes, of which 0.25 μL was mixed immediately with 0.75 μL of TRIpure LS Reagent (BioTake Co.). The resulting mixture was frozen at −80 °C until analysed.

### Primary neuron cultures

Primary neuron cultures were prepared from cerebral cortices of embryonic day 17 Sprague-Dawley rat embryos as described previously^20^. Briefly, cortices were dissected and dissociated, and the cells were cultured at 37 °C in a humidified chamber (95% air, 5% CO_2_). Cultures were used for experiments 7 to 10 days after seeding.

### RNA extraction and real-time polymerase chain reaction

Total RNA was extracted from the primary cultured cells using a commercially available kit (RNeasy Plus Mini Kit [50], QIAGEN, USA), according to manufacturer’s instructions. For the miRNA assay, total RNA (containing miRNAs) was reverse-transcribed using hairpin-loop primers designed to target the specific miRNA (miR-194-5p) at a concentration of 600 ng/μL used for complementary DNA (cDNA) synthesis PrimeScript^TM^ 1^st^ strand cDNA Synthesis Kit (TaKaRa Clontech, Cat#6110 A). Semi-quantitative real-time polymerase chain reaction (PCR) was performed using RT_2_ SYBR Green ROX qPCR Mastermix (QIAGEN, Cat. No. 330520), with U6 expression levels as an internal reference. For the mRNA assay, total RNA was reverse-transcribed with oligoDT primers at a concentration of 600 ng/μL used for cDNA synthesis PrimeScript^TM^ 1^st^ strand cDNA Synthesis Kit (TaKaRa Clontech, Cat#6110 A). TaqMan mRNA assays (Applied Biosystems Inc., Carlsbad, CA, USA) were used to quantify SUMO2 mRNA (Rn00821719-g1, Invitrogen) expression levels, in accordance with the manufacturer’s protocol. B2m (Rn00560865-m1, Invitrogen) expression levels were used as an internal reference. The CT values of the different samples were compared using the 2^−ΔΔCT^ method. Quantitative real-time PCR (qRT-PCR) was performed using 20 ng of cDNA in a 20 μL volume and the ABI 7500 System. qRT-PCR measurements were performed to obtain a mean CT value for each sample.

### *In situ* hybridization

Primary mouse cortical neurons (day 7 *in vitro*) were fixed at 4 °C overnight and treated with 5 ug/mL proteinase K for 5 min in room temperature. The cells were washed three times with phosphate-buffered saline (PBS) for 5 min per wash. Mature miR-194-5p was detected using miR-194-5p DIG-labelled LNA at both the 5′ and 3′ ends with digoxigenin probe (TCCACATGGAGTTGCTGTTAGA; Exiqon) and were prehybridized in hybridization buffer (50% formamide, 5× SSC, 200 ug/mL yeast tRNA, 200 ug/mL salmon sperm DNA, 1× Denhardt’s solution, 10% dextran sulphate (wt/vol). LNA-modified miR-194-5p were diluted to a final concentration of 40 nM in hybridization buffer, heated to 90 °C for 5 min and separately hybridized to the sections at 55 °C for 2 h in a hybridization oven (Shake N Bake, Boekel, Feasterville, PA, USA). The slides were then washed three times in 0.1× SSC (without probe) at 42 °C, followed by washing twice in 2× SSC at room temperature, then incubated in 3% H_2_O_2_ for 10 min at room temperature. They were then blocked with 1% bovine serum albumin, in 1× PBS for 1 h at room temperature and incubated with anti-digoxigenin conjugated with horseradish peroxidase (1:400, Roche Diagnostics, Mannheim, Germany) for 30 min at room temperature. The slides were washed twice with PBST and incubated for signal amplification (for the *in situ*, now labelled with horseradish peroxidase) using the TSA Cy5 kit (PerkinElmer, Waltham, MA) according to the manufacturer’s protocol. After three PBST washes (5 min each), the slides were mounted and stained with DAPI (Invitrogen). The specificity of the miR-194-5p signal in fluorescence *in situ* hybridization (FISH) experiments was confirmed when compared with a scrambled control. The scrambled probes emitted no signal in the neurons.

### MiR-194 mimic transfection and flow cytometry

The Rno-miR-194-5p mirVana^TM^ miRNA mimic ID:AM10004 (Ambion, AM17000); or Scramble ID:AM10004 (Ambion, AM17000), mixed with Lipofectamine RNAi-MAX Reagent (Invitrogen, 13778-075), were added to the primary rat neuron (7 days *in vitro*) culture medium according to manufacturer’s instructions. For the transfection efficiency assay, primary rat neurons were transfected with miR-194-5p were labelled with FAM fluorescence (miRCURY LNA Power Inhibitor of miR-194-5p *in-vivo* grade, 5′ FAM) (Exiqon, CAC00036303) for flow cytometry analysis. Briefly, attached neurons were gently trypsinized and centrifuged at 1,000 × *g* for 5 min, then labelled with Zombie VioletTM Fixable Viability Kit (Biolegend, 423113) to identify dead cells, 1 µl Zombie VioletTM reagent was added into the 1-ml culture medium, then incubated for 10 min at culture temperature. After being washed in PBS, neurons were fixed with 4% paraformaldehyde (PFA). FAM fluorescence was assayed using LSR II (BD Biosciences) and used for fluorescence acquisition. Unstained neurons (as control group) also fixed were used to determined appropriate gating parameters and voltages. The data were analysed using Flow Jo software.

### Western Blot analysis and Immunocytochemistry

Cultures were rinsed twice with PBS and the cells were collected into Pro-PREP Protein Extraction Solution (iNtRON Biotechnology, 17081). Equal amounts of protein for each sample were loaded onto 4–20% Tris-glycine gels. After electrophoresis and transferring to nitrocellulose membranes (Novex), the membranes were blocked in Tris-buffered saline containing 0.1% (vol/vol) Tween 20 and 0.2% (wt/vol) I-block (Tropix, T2015) for 90 min at room temperature. Membranes were then incubated overnight at 4 °C with following primary antibodies, anti-β -actin (1: 1,000, Sigma-Aldrich, A5441, Dorset, UK), SUMO2/3-specific antibody (1: 1,000, Abcam, ab3742, Cambridge, MA, USA). After incubation with appropriate horseradish peroxidase-conjugated secondary antibodies (GE Healthcare, NA931 (anti-mouse), or NA934 (anti-rabbit), Pittsburgh, PA, USA), the blots were developed using Pierce ECL Western Blotting Substrate Plus. Densitometric analysis was performed using Image Studio Lite software (LI-COR Biosciences) after scanning with the GE Healthcare Imager 600. For the immunocytochemistry, cells were and fixed with 4% paraformaldehyde (4% PFA) in PBS as described previously and incubated with primary antibodies against SUMO2/3 (1: 200, Abcam, ab3742). MAP2 (1: 500, Abcam, ab5392) After staining with primary antibody, fluorescent-tagged secondary antibody, nuclei were counter-stained with 4,6-diamidino-2-phenylindole. Immunostaining was analysed with a fluorescence microscope (Nikon ECLIPSE Ti-S, LosAngels, CA, USA) interfaced with a digital charge-coupled device camera and an image analysis system.

### OGD, re-oxygenation, and hypothermia OGD

OGD experiments were performed using a specialized, humidified chamber (Heidolph, incubator 1000, Brinkmann Instruments) maintained at 37 °C, which contained an anaerobic gas mixture (90% N_2_, 5% H_2_, and 5% CO_2_). For hypothermia OGD, the OGD chamber was set to room temperature (25 °C). To initiate OGD, culture medium was replaced with deoxygenated, glucose-free DMEM (Life Technologies, USA). After a 2 h challenge, cultures were removed from the anaerobic chamber, and the OGD solution in the cultures was replaced with maintenance medium. Neurons were then allowed to recover for 18 h in a regular incubator before the neurotoxicity assay.

### Determination of cell viability

Cell viability was quantified using the Cell Counting kit-8 (CCK-8, Dojindo) according to manufacturer’s instructions. CCK-8 solution (10 μL) was added to each well of the plate, and the cells were incubated at 37 °C for 2 h. The optical density at a wavelength of 450 nm was measured using a microplate reader. The relative assessments of neuronal injury were normalized by comparison with control neurons, in which cell survival was defined to be 100%. Cell viability was also assessed after staining of neuron cultures with propidium iodide (PI) to distinguish between living and dead cells (0.001 mg/mL for 5 minutes with subsequent rinsing) and five images per well were taken and quantified as ratios versus all neurons under bright light.

### Statistical analysis

All values are presented as mean ± standard deviation(SD) of at least three independent experiments. For measurement data, multiple comparisons were evaluated using one-way ANOVA followed by the Tukey-Kramer test for pair-wise comparisons between all groups. A repeated-measures two-way ANOVA was followed by Bonferroni test. An unpaired t-test was used only for two-group comparisons. Differences with P < 0.05 was considered to be statically significant.

## Electronic supplementary material

Supplementary-Figure.

Supplementary-Table.

## Data Availability

The data are available from the corresponding author on reasonable request.
