# Supplementary material for: MicroRNA-194-5p Levels Decrease during Deep Hypothermic Circulatory Arrest
Source: Sci Rep. 2018 Sep 19;8:14044. doi: 10.1038/s41598-018-32426-x (PMC6145897; doi:10.1038/s41598-018-32426-x)

***Original paper***

**MicroRNA-194-5p Levels Decrease during Deep Hypothermic
Circulatory Arrest**

**Running head:** miRNA levels in response to DHCA

Xiaohua Wang, MD PhD, ^1,2,3^, Zerong You, PhD^5^, Guoguang Zhao, MD, * ^2,3,4^, Tianlong Wang, MD, * ^1,2,3^

^1^ Department of Anesthesiology, Xuanwu Hospital, Capital Medical University, Beijing 100053, China. ^2^ Institute of Geriatrics, Beijing, China. ^3^ National Clinical Research Center for Geriatric Disorders, Beijing, China. ^4^ Department of Neurosurgery, Xuanwu Hospital, Capital Medical University, Beijing 100053, China.^5^ Department of Anesthesiology, MGH, Harvard medical school.

*Co-corresponding authors at: Tianlong Wang, Department of Anesthesiology, Xuanwu Hospital, Capital Medical University, Beijing 100053, China. E-mail address: w_tl5595@yahoo.com. Guoguang Zhao, Department of Neurosurgery, Xuanwu Hospital, E-mail address: ggzhao@vip.sina.com

**Supplementary-Figure.**

(A) MAP2 and DAPI staining reflected the cultured neurons status under normal condition in the control group.

(B) SUMO2 expression in the neurons.


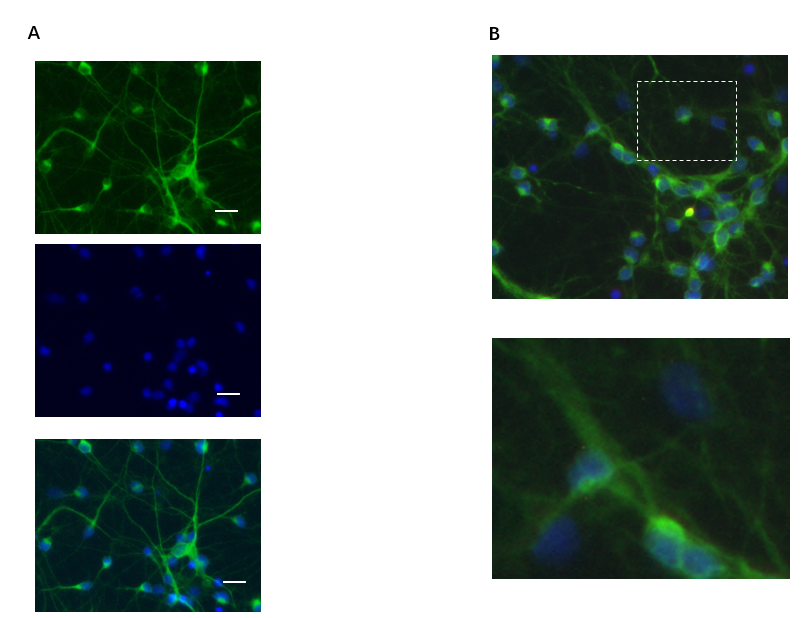

Supplement: Supplementary file 1 — Supplementary-Figure. [file 41598_2018_32426_MOESM1_ESM.doc]
