# Supplementary material for: MicroRNA-194-5p Levels Decrease during Deep Hypothermic Circulatory Arrest
Source: Sci Rep. 2018 Sep 19;8:14044. doi: 10.1038/s41598-018-32426-x (PMC6145897; doi:10.1038/s41598-018-32426-x)
Supplement: Supplementary file 2 — Supplementary-Table. [file 41598_2018_32426_MOESM2_ESM.doc]

***Original paper***

**Title page**

**MicroRNA-194-5p Levels Decrease during Deep Hypothermic Circulatory Arrest**

**Running head:** miRNA levels in response to DHCA

Xiaohua Wang, MD PhD, ^1,2,3^, Zerong You, PhD^5^, Guoguang Zhao, MD, * ^2,3,4^, Tianlong Wang, MD, * ^1,2,3^

^1^ Department of Anesthesiology, Xuanwu Hospital, Capital Medical University, Beijing 100053, China. ^2^ Institute of Geriatrics, Beijing, China. ^3^ National Clinical Research Center for Geriatric Disorders, Beijing, China. ^4^ Department of Neurosurgery, Xuanwu Hospital, Capital Medical University, Beijing 100053, China.^5^ Department of Anesthesiology, Massachusetts General Hospital and Harvard Medical School, Charlestown, Massachusetts 02148, USA.

*Co-corresponding authors at: Tianlong Wang, Department of Anesthesiology, Xuanwu Hospital, Capital Medical University, Beijing 100053, China. E-mail address: w_tl5595@yahoo.com. Guoguang Zhao, Department of Neurosurgery, Xuanwu Hospital, E-mail address: ggzhao@vip.sina.com

**Supplementary-Table.**

The different expression of microRNAs undergoing Deep hypothermic circulatory arrest (DHCA) in piglet model.

| Downregulated microRNAs | miR-194-5p; miR-670; miR-3178; miR-541; miR-454; miR-150; miR-210; miR-1207-3p; miR-130b*; miR-940; miR-135a*; miR-1281; miR-1224-5p; miR-216b; miR-383; miR-122; miR-720; miR-1280; miR-10b; miR-891a; miR-200c; miR-200a; |
| --- | --- |
| Upregulated microRNAs | miR-27a*; miR-23a*; miR-149; miR-218-2*; miR-363*; miR-182; miR-663; miR-1910; miR-638; miR-143*; miR-218; miR-206 |

The miRNAs were significantly differential expressions between sham and treatment groups. Each group have three biological replicates. Colors represent expression change: red, up-regulation; green, down-regulation.
